# The Immunogenetic Landscape of Allergic Rhinitis: from Cellular Effectors to Gene Regulation and Targeted Therapies

**DOI:** 10.7150/ijbs.126788

**Published:** 2026-01-01

**Authors:** Xu Zhang, Zhiqiang Zhang, Qian Peng, Xinyu Huang, Mengyuan Liu, Daoming Bai, Rui Yang, Yun Zhang, Chunping Yang

**Affiliations:** Department of Otorhinolaryngology Head and Neck Surgery, The Second Affiliated Hospital, Jiangxi Medical College, Nanchang University, Nanchang, Jiangxi Province, China.

**Keywords:** type 2 inflammation, Th2 Cells, ILC2s, epithelial alarmins, GATA3, genetic susceptibility, targeted biologics

## Abstract

Allergic Rhinitis (AR) is a highly prevalent type 2 inflammatory disease driven by a complex immunogenetic background. This review aims to systematically delineate the immunogenetic landscape of AR, elucidating the complete knowledge chain from macroscopic cellular interactions and microscopic molecular regulation to precision targeted therapies. The article first dissects the two core immune axes driving the pathological process of AR: one is the classic adaptive immune pathway, centered on Th2 cells, which mediates IgE production, eosinophil infiltration, and mucus hypersecretion through the secretion of cytokines such as IL-4, IL-5, and IL-13; the other is the innate immune initiation pathway, in which nasal epithelial cells act as "sentinels" by releasing "alarmins" like TSLP and IL-33, leading to the rapid activation of type 2 innate lymphoid cells (ILC2s). The review then delves into the sophisticated signaling networks that regulate these immune responses, with a particular focus on the classic IL-4/STAT6/GATA3 signaling axis and its negative regulatory mechanisms. Building on this, the article further elaborates on the genetic susceptibility architecture of AR, highlighting key risk loci identified by genome-wide association studies (GWAS), such as variants in antigen presentation genes (HLA), epithelial barrier genes (FLG), and genes related to cytokine signaling pathways. To connect genetics with the environment, this review systematically summarizes epigenetic regulatory mechanisms, including DNA methylation, histone modifications, and microRNAs (miRNAs), and discusses the long-range immunomodulatory effects of nasal and gut microbiota dysbiosis on AR via the "gut-nasal axis". Finally, from a translational medicine perspective, the article demonstrates how a profound understanding of these pathophysiological mechanisms has successfully spurred the development of highly effective targeted biologics, such as omalizumab (targeting IgE), dupilumab (targeting the IL-4Rα receptor, thus blocking IL-4/IL-13 signaling), and tezepelumab (targeting TSLP). This review integrates the latest multidimensional research advances in immunology, genetics, epigenetics, and microbiome studies of AR, providing a comprehensive theoretical framework for understanding its complex pathogenesis and for the development of future personalized treatment strategies.

## Introduction: The Cellular Microenvironment of Type 2 Inflammation in the Upper Airway

At the core of Allergic Rhinitis (AR) lies a dysregulated type 2 immune response within the upper airway mucosa. Its pathological process involves a complex network composed of various immune cells, structural cells, and the molecular mediators they secrete. Conventionally, this process is understood to be predominantly orchestrated by T helper 2 (Th2) cells of the adaptive immune system. Following the capture and presentation of allergens by antigen-presenting cells, naïve CD4+ T cells differentiate into Th2 cells under a specific cytokine milieu. These Th2 cells then initiate and direct the entire allergic cascade, centered on IgE class-switching, through the secretion of a series of signature cytokines, such as IL-4, IL-5, and IL-13.

However, as immunological research has advanced, it is now recognized that the innate immune system plays an equally vital and indispensable role in the initiation and amplification of AR. As the first line of defense against the external environment, nasal epithelial cells are not merely passive physical barriers but active immune "sentinels." Upon stimulation by allergens and other triggers, epithelial cells release "alarmins" such as Thymic Stromal Lymphopoietin (TSLP), Interleukin-33 (IL-33), and Interleukin-25 (IL-25). These signaling molecules can rapidly activate innate immune cells. Among them, type 2 innate lymphoid cells (ILC2s), acting as "first responders" to inflammation, can react swiftly to these alarmins without prior sensitization. They produce large quantities of type 2 cytokines, thereby initiating the inflammatory process even before the adaptive immune response is established.

**Figure 1:**
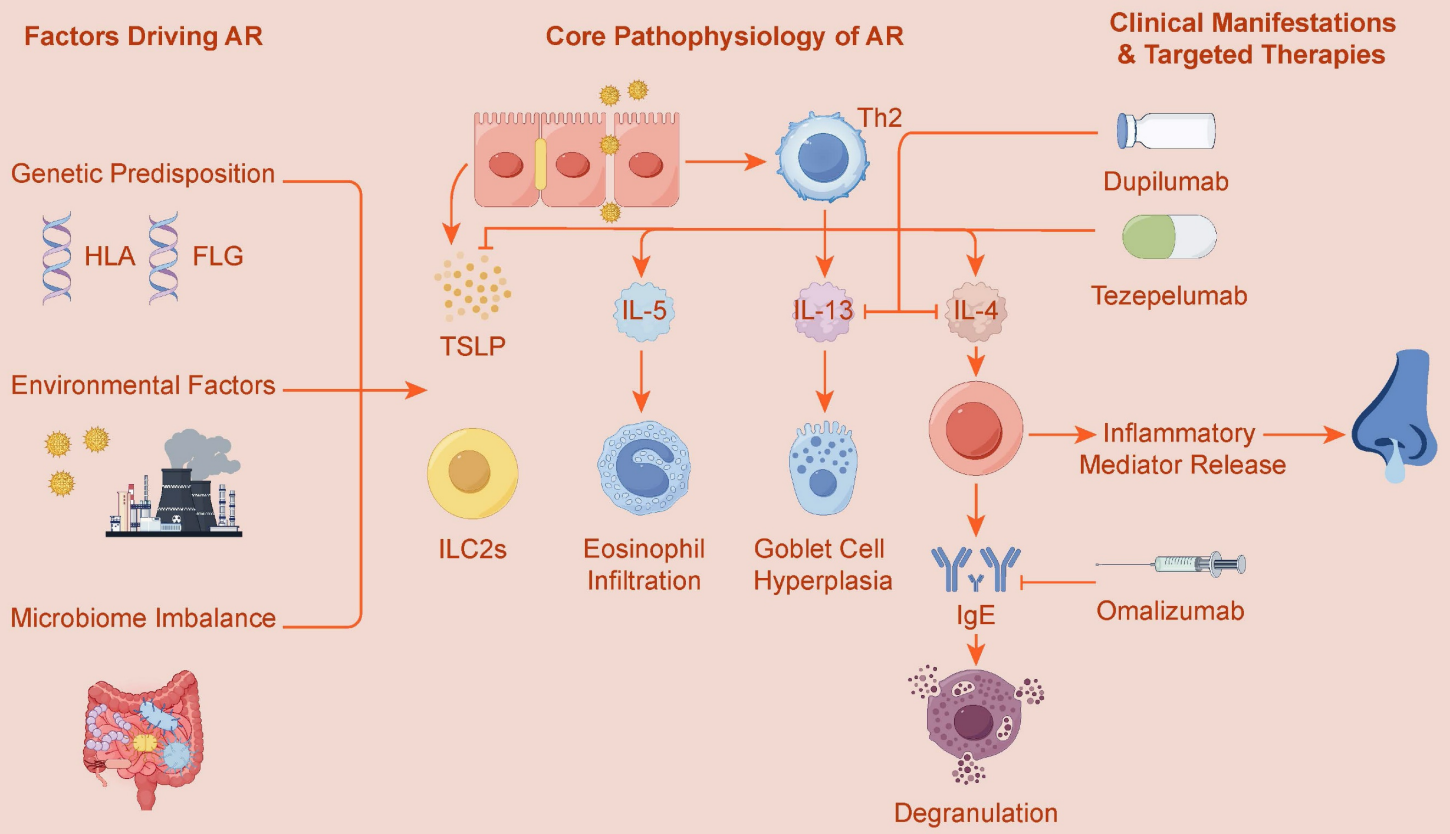
Core Immunopathology and Targeted Therapies of Allergic Rhinitis (AR). This figure illustrates the pathogenesis of Allergic Rhinitis (AR), driven by genetic predisposition (e.g., HLA, FLG genes) and environmental factors, including microbiome imbalance. The core pathology centers on a type 2 inflammatory response, involving both innate immunity, where alarmins like TSLP activate ILC2s, and adaptive immunity, centered on Th2 cells. These pathways lead to the release of key cytokines (IL-4, IL-5, IL-13), which subsequently cause IgE production, eosinophil infiltration, and goblet cell hyperplasia. This mechanistic understanding has enabled the development of targeted therapies such as Omalizumab (anti-IgE), Dupilumab (anti-IL-4/IL-13), and Tezepelumab (anti-TSLP).

Therefore, the cellular microenvironment of AR is a dynamic system characterized by tight collaboration and mutual reinforcement between innate and adaptive immunity. The activation, amplification, and regulation of these two immune axes depend on a sophisticated network of cytokines, transcription factors, and signaling pathways, and are profoundly influenced by multiple factors including genetic susceptibility, epigenetic modifications, and the microbiome. Ultimately, these complex interactions collectively shape the clinical phenotype of AR. Figure 1 provides an overview of the core pathophysiology driving AR, highlighting the interplay between these innate and adaptive pathways. This review aims to systematically elucidate the complete immunogenetic landscape of AR, starting from key cellular effectors, and progressively delving into molecular signaling, gene regulation, and multi-omics interactions, finally focusing on how this fundamental knowledge can be translated into precise targeted therapeutic strategies.

## The Adaptive Immune Axis: Th2 Cells and the Classic Allergic Cascade

In allergic rhinitis (AR), Th2 cells are the core of the type 2 immune response. When an allergen is presented by antigen-presenting cells (APCs) to naïve CD4+ T cells, these T cells differentiate into Th2 cells under the induction of cytokines like interleukin-4 (IL-4), thereby initiating the entire allergic cascade^1^. The differentiation of Th2 cells is governed by the master transcription factor GATA3, whose expression is regulated by the IL-4/JAK/STAT6 signaling pathway^2^, ^3^. The number of Th2 cells is significantly increased in AR patients, leading to a shift in the Th1/Th2 balance towards Th2, which is a key pathogenic factor in allergic diseases^4^.

Activated Th2 cells secrete a variety of cytokines, prominently including IL-4, IL-5, and IL-13, which collaboratively drive the complex pathological processes of AR^2^, ^5^.

**Figure 2:**
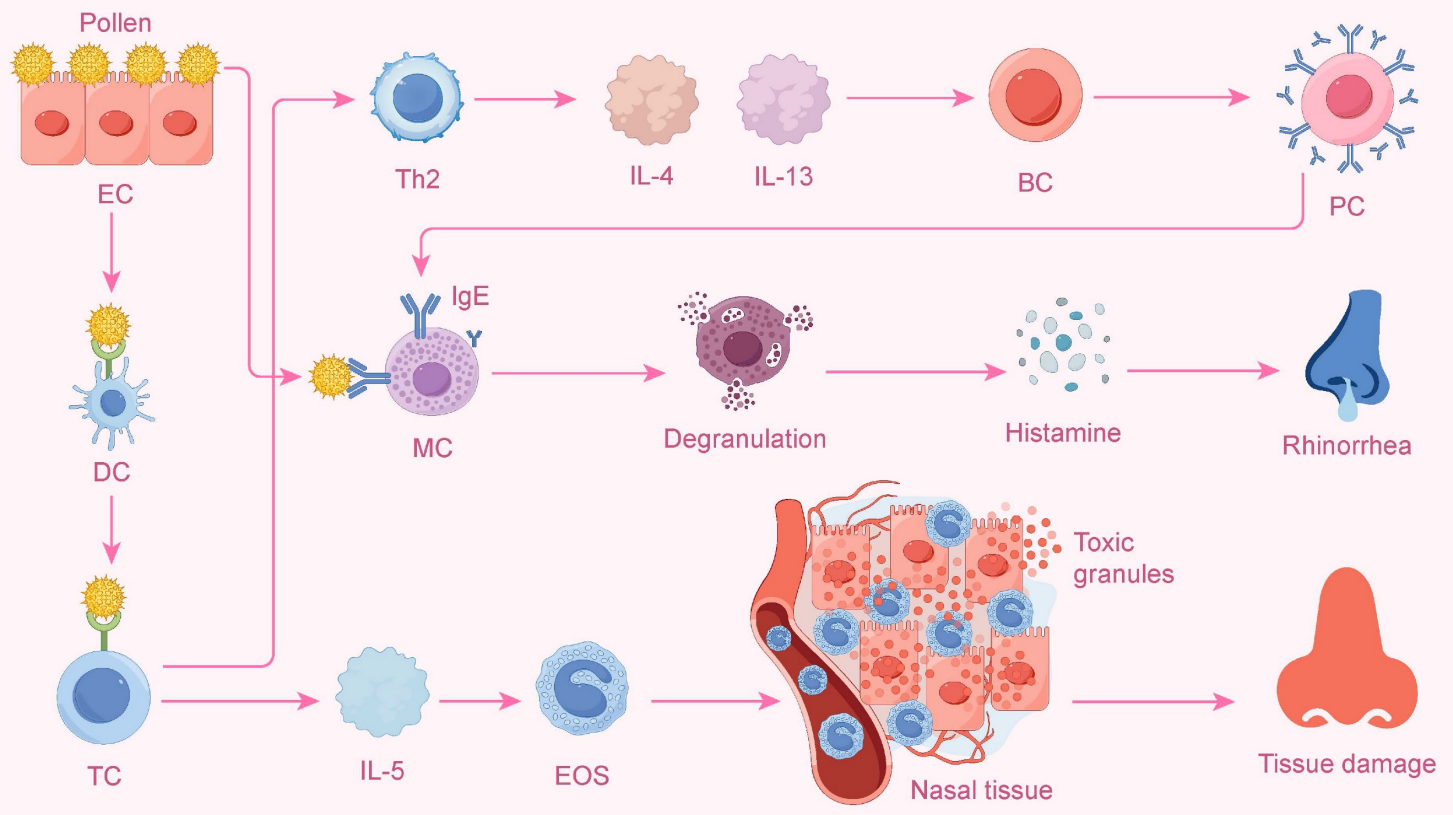
The Th2-Mediated Allergic Cascade in Allergic Rhinitis. This diagram shows the classic allergic cascade initiated by allergens like pollen. A dendritic cell (DC) presents the allergen to a T cell (TC), inducing its differentiation into a Th2 cell. The Th2 cell then orchestrates two main pathways. First, it releases IL-4 and IL-13, which cause B cells (BC) to become plasma cells (PC) that produce IgE. IgE sensitizes mast cells (MC), and subsequent allergen exposure triggers their degranulation, releasing histamine and causing symptoms like rhinorrhea. Second, the Th2 cell secretes IL-5, which activates eosinophils (EOS). These eosinophils infiltrate nasal tissue and release toxic granules, leading to tissue damage.

To provide a more intuitive illustration of this classic process, Figure 2 depicts the complete cascade triggered by allergens and dominated by Th2 cells, ranging from upstream cell differentiation to downstream inflammatory effects.

### IL-4: Driving Th2 Cell Differentiation and Inducing B-Cell Production of Allergen-Specific IgE Antibodies

As a critical driver for initiating and amplifying the allergic cascade, IL-4 plays a central role in the activation of the Th2 immune axis^6^, ^7^. It not only drives the differentiation of naïve T cells toward a Th2 phenotype by activating the STAT6 signaling pathway and upregulating the master transcription factor GATA-3, but it also directly induces immunoglobulin class switching in B cells to produce allergen-specific IgE^6^, ^8^. In mouse models of AR, the allergic group exhibits a typical Th2-dominant response, with significantly upregulated expression of GATA-3 and STAT6, while the key Th1 transcription factor T-bet is suppressed, thereby disrupting the Th1/Th2 balance of the immune system^6^.

The direct action of IL-4 on B cells is the decisive step for IgE production, as it promotes B-cell class switch recombination (CSR) to IgE^7^, ^9^. Recent studies have found that type 2 T follicular helper (Tfh2) cells are a major source of IL-4. In AR patients, Tfh2 cells, by secreting IL-4, can more effectively induce memory B cells to express CD23, an important molecule for IgE synthesis and antigen presentation^8^, ^10^. In animal models, blocking IL-4 or depleting CD4+ T cells effectively suppresses serum IgE production, further confirming the central position of IL-4 in IgE-mediated allergic reactions^6^, ^11^.

### IL-5: Recruiting and Activating Eosinophils, Leading to Late-Phase Reaction and Chronic Inflammation

In the Th2-driven allergic reaction, interleukin-5 (IL-5) is the core effector molecule that mediates the late-phase reaction and chronic inflammation^12^. It is not only critical for eosinophil activation but also induces precursor formation and supports eosinophil survival. Its primary function is to recruit and activate eosinophils. The nasal allergen challenge (NAC) model visually demonstrates this process: following the challenge, intranasal IL-5 levels remain elevated, peripheral blood eosinophils decrease, and correspondingly, eosinophils in the nasal mucosal tissue increase, confirming that IL-5 drives their migration from the blood to the site of inflammation^13^.

Eosinophil infiltration is a key pathological feature of the late-phase reaction^14^, and its symptoms are closely associated with a concurrent increase in IL-5 and eosinophils^15^. IL-5 is not only responsible for recruitment but can also "prime" eosinophils for activation by co-stimulatory factors; *in vitro* experiments have confirmed that an anti-IL-5 antibody can effectively inhibit their activation^16^. The sustained activation of the IL-5/eosinophil axis is critical for the transition of AR to chronic inflammation^15^. Consequently, pharmacological interventions that reduce IL-5 levels can significantly decrease eosinophil infiltration and improve clinical symptoms, which conversely validates the central role of this axis in the pathology of AR^14^, ^17^. It is worth noting, however, that eosinophil functions are not exclusively pathological; they can also have non-pathological aspects in tissue homeostasis and host defense.

### IL-13: Causing Mucus Hypersecretion and Tissue Remodeling

Interleukin-13 (IL-13), another key Th2 cytokine, is a primary driver of mucus hypersecretion and tissue remodeling in AR^18^, ^19^. IL-13 can directly stimulate airway epithelial cells, inducing them to produce and secrete mucus, particularly the key mucin MUC5AC, which is the major gel-forming mucin responsible for the viscoelastic properties of airway mucus^19^-^21^.

At the cellular level, IL-13 increases the sources of mucus secretion by promoting goblet cell hyperplasia, which is the core mechanism by which it causes mucus hypersecretion^22^, ^23^. Goblet cell hyperplasia, along with pathological changes like eosinophil recruitment and subepithelial fibrosis, collectively constitutes the process of airway tissue remodeling mediated by IL-13^18^, ^21^. From a molecular standpoint, IL-13 primarily exerts its biological effects by activating the phosphorylation of the STAT6 signaling pathway^21^, ^24^.

In summary, Th2 cells and their secreted cytokines, such as IL-4, IL-5, and IL-13, collectively coordinate the complete pathological chain of allergic rhinitis. Recent studies have further unveiled a multi-dimensional regulatory network for Th2 cell differentiation, including factors such as long non-coding RNAs^3^, ^25^, cellular metabolic states^26^, and endoplasmic reticulum stress^27^, all of which provide new perspectives for understanding the pathogenesis of AR.

To systematically summarize the key roles, upstream and downstream regulatory relationships, and specific mechanisms of the core components of the Th2 immune axis in the pathological process of allergic rhinitis, we have compiled the information above into Table 1 for a multi-dimensional comparative analysis.

## The Innate Immune Axis: Epithelial Cells and ILC2s as Key Initiators

### Epithelial Cells as Immune Sentinels and Their Release of "Alarmins" (TSLP, IL-33, IL-25)

As the first line of defense against the environment, nasal epithelial cells play a crucial "sentinel" role in allergic rhinitis (AR)^28^, ^29^. They actively respond to allergens by releasing "alarmins" such as thymic stromal lymphopoietin (TSLP), interleukin-25 (IL-25), and interleukin-33 (IL-33), which initiate and amplify the type 2 immune response, constituting the initial signal of the allergic cascade^30^.

Studies have confirmed that in patients with AR and related eosinophilic chronic rhinosinusitis (ECRS), epithelial cells are the main source of alarmins, and their expression levels are significantly upregulated^30^, ^31^. For example, the expression of TSLP, IL-25, and IL-33 in the epithelium of nasal polyps from ECRS patients is significantly higher than in control groups, and their levels positively correlate with the clinical severity of the disease (such as CT scores and eosinophil counts), indicating that these epithelial-derived alarmins are core factors driving the disease's pathological process^30^.

Common inhaled allergens, such as house dust mites (Der p 1), fungi, and pollen, are key stimuli for triggering the release of alarmins^30^, ^32^, ^33^. In vitro experiments show that allergens can induce epithelial cells to produce alarmins; for instance, the house dust mite allergen (Der p 1) can induce the release of IL-33 in a dose- and time-dependent manner. The induction mechanism is related to the biological activity of the allergens themselves; for example, the protease activity and NADPH oxidase activity of pollen play an important role in inducing the release of IL-25^33^.

Furthermore, the nasal epithelial cells of AR patients exhibit intrinsic dysfunction, placing them in a "pre-activated" state. Even without allergen stimulation, the baseline mRNA levels of TSLP in the epithelial cells of AR patients are significantly higher than in healthy individuals^29^. This functional abnormality is associated with defects in epithelial protective mechanisms. For example, the expression of endogenous protease inhibitors (EPIs) is reduced in the epithelial cells of ECRS patients, which amplifies allergen-induced alarmin release^34^. Concurrently, the abnormal activation of upstream signaling pathways, such as Notch-1, can also sustain the high expression of IL-33^35^.

In summary, nasal epithelial cells are active immune sentinels. In AR, their intrinsic functional defects and weakened protective mechanisms lead to an excessive release of alarmins like TSLP, IL-33, and IL-25 upon allergen stimulation. Thus, they act as key initiators, triggering the downstream type 2 inflammatory immune response. Furthermore, alarmins can directly interact with DCs to shape the later immune response. These epithelial-derived signals directly act on downstream immune cells, among which type 2 innate lymphoid cells (ILC2s), as the "first responders" of inflammation, constitute the next critical link in the immune cascade.

### ILC2s as "First Responders" of Inflammation, Rapidly Producing IL-5 and IL-13

As key effector cells of the innate immune system, Group 2 innate lymphoid cells (ILC2s) play the role of "first responders" in the initiation phase of allergic inflammation. Unlike adaptive immune cells that require antigen presentation and clonal expansion, ILC2s can mount a rapid, antigen-independent response directly to epithelial-derived alarmins, thereby promptly initiating and amplifying the type 2 immune response^36^.

The activation of ILC2s is a central event in the "epithelial cell-ILC2" innate immune axis. Upon stimulation by allergens (eg, house dust mites, fungi) or pathogen-derived products (eg, papain), the nasal mucosal epithelium rapidly releases alarmins such as IL-25, IL-33, and thymic stromal lymphopoietin (TSLP)^37^, ^38^. ILC2s express corresponding receptors for these alarmins on their surface, such as ST2 (IL-33R), allowing for their swift activation. This activation mechanism is particularly evident in the nasal tissues of patients with allergic rhinitis (AR), where the presence of ILC2s can be observed *in situ*, and their numbers correlate significantly with the levels of IL-33, TSLP, and IL-25^39^, ^40^. Functional evidence from animal models confirms this, showing that in Rag2-/- mice lacking T and B cells, activation of ILC2s alone is sufficient to induce nasal inflammatory symptoms, highlighting their independent role as innate "first responders"^41^. Once activated, ILC2s quickly adjust their metabolic state, for instance, by increasing fatty acid uptake, storage, and oxidation, to support their highly efficient effector functions^42^.

The primary function of ILC2s is the rapid and abundant production of the signature type 2 cytokines, IL-5 and IL-13^38^, ^43^. IL-5 is mainly responsible for the recruitment and activation of eosinophils, while IL-13 has broader effects, including the promotion of epithelial hyperplasia and mucus secretion, which together drive the key pathological features of allergic rhinitis^37^, ^41^. In a mouse model of nasal allergy, ILC2-derived IL-13 was confirmed to be a critical mediator in exacerbating both nasal epithelial thickening and eosinophilia^41^.

In summary, by directly responding to epithelial alarmin signals, ILC2s act as "first responders" to rapidly produce IL-5 and IL-13, thereby quickly initiating and establishing the foundation for the type 2 inflammatory response in allergic rhinitis^37^, ^38^. Importantly, the early inflammatory environment established by this innate axis serves to instruct the subsequent adaptive response.

To systematically summarize the initiation process of the innate immune axis in allergic rhinitis, we have organized the "sentinel" role of epithelial cells and the "first responder" function of ILC2s, along with their related key molecules and signaling pathways, into Table 2. This allows for a clear understanding of the various stages and core elements of this process.

### The Transcriptional and Signaling Network Regulating Type 2 Immunity

The initiation and regulation of the type 2 immune response depend on a sophisticated network composed of cytokines, transcription factors, and signaling pathways. At the core of this network is the classic IL-4/STAT6/GATA3 signaling axis, which not only directly drives the differentiation of Th2 cells but also, through interactions with other regulatory elements, collectively determines the intensity and direction of the type 2 immune response.

**Figure 3:**
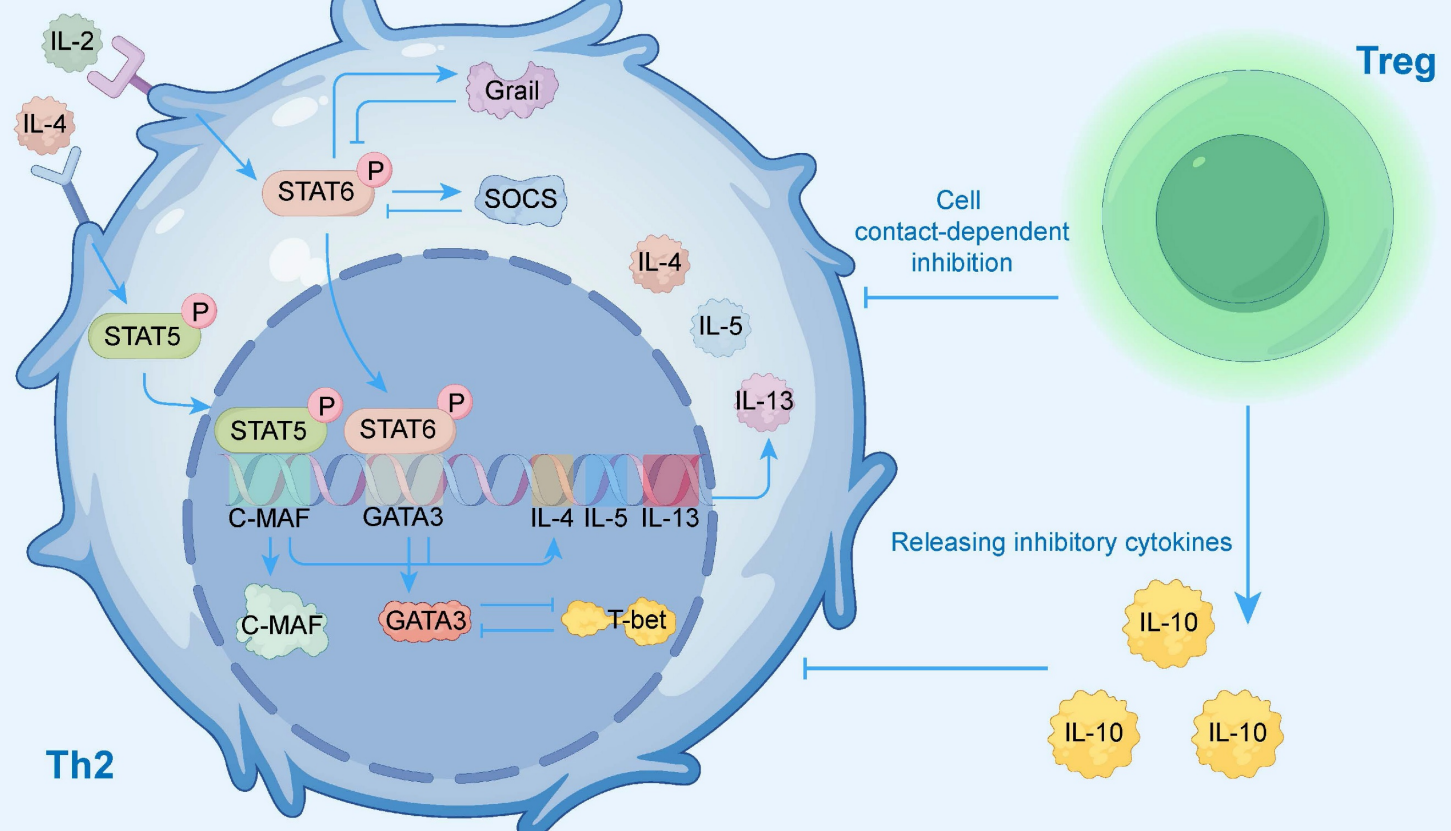
Signaling and Transcriptional Regulation of Th2 Cells. This diagram illustrates the complex network regulating Th2 cell function. Key activating pathways are initiated by external cytokines: Interleukin-4 (IL-4) activates the STAT6 pathway, and Interleukin-2 (IL-2) activates the STAT5 pathway. These signals induce the master transcription factors GATA3 and C-MAF, which drive the expression of effector cytokines IL-4, IL-5, and IL-13. This network is tightly controlled by multiple negative feedback mechanisms. Internally, the STAT6 pathway induces inhibitors like SOCS and Grail, while the transcription factor T-bet antagonizes the Th2 program. Externally, regulatory T cells (Tregs) suppress Th2 cells through both direct cell contact-dependent inhibition and the release of inhibitory cytokines like IL-10.

To visually present this complex regulatory network, Figure 3 illustrates the key activating and inhibitory signaling pathways within Th2 cells, as well as the external regulation exerted by regulatory T cells (Tregs), providing readers with an overall conceptual framework. The activation of these signaling pathways (e.g., IL-4R, TSLP) is central to AR pathology, and as the next section will detail, genetic variations within those pathway components are a key determinant of individual susceptibility.

### The IL-4/STAT6/GATA3 Axis as the Core Pathway for Th2 Differentiation

The differentiation of naïve CD4+ T cells into Th2 cells is primarily driven by the classic IL-4/STAT6/GATA3 signaling axis^44^, ^45^. This process begins with the binding of IL-4 to its receptor (IL-4R). The stability of this binding can be regulated by the cell surface molecule CD44v5, which prevents the degradation of IL-4Rα by interacting with it, thereby ensuring the effective initiation of the signal^46^.

Upon signal activation, STAT6 is recruited and phosphorylated, then enters the nucleus, where it acts as a key signal transducer to induce the expression of the master transcription factor GATA3^47^, ^48^. GATA3 is the "master regulator" of Th2 cell differentiation, responsible for initiating the transcriptional program of Th2 signature cytokines (IL-4, IL-5, IL-13) and suppressing the Th1 pathway^45^. However, GATA3 does not function in isolation; the expression of many Th2-specific genes depends on the synergistic regulation of both GATA3 and STAT6^48^. The IL-4/STAT6 axis also fine-tunes the Th2 response by regulating other transcription factors. For instance, this pathway indirectly promotes Th2 differentiation by inhibiting the suppressive isoforms of TCF-1^49^, while simultaneously inducing the expression of SATB1, which is further involved in regulating the transcription of cytokines like IL-5^50^.

To maintain immune homeostasis, this signaling axis is under strict negative feedback control. The IL-4/STAT6 pathway itself induces the expression of the E3 ubiquitin ligase Grail, which in turn targets and degrades STAT6, forming a classic negative feedback loop^47^. Additionally, the cytokine-inducible SH2-containing protein (CIS) can also limit the Th2 response by inhibiting STAT6 signaling^51^. The latest research has also found that dysregulation of organelle homeostasis can trigger this pathway. For example, mitochondrial DNA leakage caused by BLOC1S1 deletion can enhance Th2 polarization by activating STING-NF-κB signaling, which ultimately promotes STAT6 phosphorylation and GATA3 expression^52^.

### STAT6-Independent Pathways (Notch, IL-2/STAT5)

Although the IL-4/STAT6 signaling axis is crucial for type 2 immunity, the differentiation, function, and migration of Th2 cells are also regulated by key STAT6-independent signaling pathways, with the Notch signaling and IL-2/STAT5 pathways being particularly prominent.

In the Th2 immune response, Notch signaling is not the initial signal for differentiation but rather acts as a critical "licensing" signal that regulates the function and behavior of mature Th2 cells. Its core role in allergic airway inflammation is to promote the egress of Th2 cells from draining lymph nodes to effector tissues such as the lungs^53^. This function is achieved by upregulating the KLF2/S1PR1 axis, independent of the classic Th2 transcription factor GATA3^53^. The activation of Notch signaling is tightly regulated by dendritic cells (DCs). The transcription factor KLF2 within DCs negatively regulates the expression of the Notch ligand Jagged2 via HIF-1α^54^, while the E3 ubiquitin ligase Mind bomb-1 (Mib1) serves as a necessary molecular switch for the ligand to gain functional activity and effectively activate Notch signaling in T cells^55^.

The IL-2/STAT5 pathway is another key STAT6-independent route for Th2 differentiation. In human CD4+ T cells, IL-2 directly induces the expression of C-MAF, a transcription factor crucial for Th2 differentiation, by activating STAT5^56^. This pathway is also synergistically amplified by other transcription factors; for instance, the Ikaros family protein Eos can form a complex with STAT5, enhancing its phosphorylation level and transcriptional activity, thereby cooperatively promoting the expression of Th2-related genes^57^. To prevent excessive immune responses, this pathway is also subject to sophisticated negative feedback regulation. The CIS and SOCS2 proteins of the suppressor of cytokine signaling (SOCS) family can both control the intensity of the Th2 response by inhibiting the phosphorylation of STAT5 and STAT6, forming a critical negative feedback loop^58^, ^59^.

### GATA3 as the Common Core Transcription Factor for Th2 Cells and ILC2s

The transcription factor GATA3 is the common core regulator that drives the differentiation, maintenance, and function of both Th2 cells and ILC2s, the two key effector cells of type 2 immunity^60^-^62^. GATA3 not only directly regulates the expression of various type 2 cytokines (such as IL-4, IL-5, IL-13)^63^, ^64^, but its own expression is also controlled by a multi-layered, highly context-dependent, and cell-type-specific complex network.

The expression of GATA3 is subject to cell-specific regulation by different distal enhancers. For example, the Gata3 +674/762 region and the G3SE super-enhancer are crucial for the development of ILC2s^61^, ^62^, whereas an asthma-associated mG900 region is key for Th2 cell differentiation^65^. The upstream transcriptional network also exhibits high cell specificity. In lung Th2 cells, an IL-10-STAT3-Blimp-1-Bcl6 signaling axis has been shown to indirectly promote GATA3 expression^63^. In contrast, the transcription factor BACH2 is a necessary positive regulator in ILC2s but plays an inhibitory role in Th2 cells^66^. Furthermore, the dosage of GATA3 expression is also critical; for instance, lower levels of GATA3 in skin ILC2s allow for greater plasticity^67^. Studies have also shown that GATA3 expression in mature type 2 lymphocytes is not dependent on self-positive feedback, as its gene transcription activity can be maintained even if the GATA3 protein loses its function^64^.

### The Negative Regulatory Network of Type 2 Immunity

To maintain immune homeostasis, the type 2 immune response is strictly controlled by various negative regulatory mechanisms. In addition to the classic transcription factor T-bet, which can directly inhibit the Th2 cell program^68^, a regulatory network centered on regulatory T cells (Tregs) is also crucial, effectively suppressing allergic inflammation through multiple mechanisms.

The suppressive function of Tregs depends on the precise regulation of various molecules within the cells. The transcription factor BACH2 acts as a key checkpoint, directly inhibiting type 2 inflammatory immunity^69^. At the post-transcriptional level, members of the microRNA family miR-15/16 are essential for maintaining the high suppressive capacity of Tregs, and their absence exacerbates allergic inflammation^70^. Additionally, the cell signaling protein DOCK8 is indispensable for maintaining the stability and function of Tregs; its deficiency leads to Treg dysfunction, thereby amplifying the type 2 immune response to allergens^71^.

Simultaneously, the suppressive function of Tregs requires "licensing" and "activation" by external signals. The prostaglandin I₂ (PGI₂) signaling pathway "licenses" the suppressive function of Tregs and prevents their reprogramming into pathogenic Th2-like cells in an inflammatory environment^72^. The anti-inflammatory cytokine IL-37 can alleviate allergic inflammation by increasing the number of Tregs and the secretion of IL-10^73^. Interestingly, the classic type 2 inflammation driver TSLP can also form a negative feedback loop; it acts directly on Tregs to maintain their lineage stability, thereby limiting the excessive development of allergic inflammation^74^. Furthermore, the IL-6 signaling pathway provides a T-bet-independent mechanism for Th2 inhibition by inducing SOCS3 to suppress the IL-2 signaling required for Th2 cell differentiation^68^.

The regulatory effects of Tregs are multi-targeted and nuanced. They not only inhibit Th2 cells but can also directly attenuate the activation of type 2 innate lymphoid cells (ILC2s)^75^. Their regulation is not entirely suppressive; for example, soluble CTLA-4 (sCTLA-4) secreted by Tregs can selectively inhibit type 1 immunity while permitting type 2 immune responses, which may facilitate subsequent tissue repair^76^. Therefore, when Treg function is impaired due to intrinsic defects or is suppressed by external molecules (such as BAFF), this precise negative regulatory balance is disrupted, directly leading to the onset and exacerbation of allergic inflammation^77^.

In summary, a network composed of the core IL-4/STAT6/GATA3 axis, STAT6-independent pathways, and a negative regulatory mechanism co-dominated by T-bet and Tregs, involving intrinsic molecular programs and precisely regulated by external signals, collectively constitutes the key barrier for regulating the type 2 immune response.

To systematically organize the complex regulatory network described above, Table 3 provides a detailed summary of the key molecules, cells, and their functions within the core signaling axis, STAT6-independent pathways, the crucial role of GATA3, and the multi-layered negative regulatory mechanisms that govern the type 2 immune response.

## The Genetic Architecture of Allergic Rhinitis Susceptibility

Allergic rhinitis (AR) is a complex disease with high heritability^78^, ^79^. Genome-wide association studies (GWAS) and their derived new analytical strategies, such as multi-trait analysis of GWAS (MTAG), have become central tools for identifying its polygenic background and risk loci^80^, ^81^.

### Identification of AR Risk Loci by GWAS

GWAS has successfully identified multiple AR susceptibility loci, many of which exhibit specificity or pleiotropy across different populations and allergic traits.

In Chinese and East Asian populations, several studies have discovered new risk loci. For example, a genome-wide gene-based association study (GWGAS) identified ZNF608 as a key risk gene for dust mite-induced AR^80^. Using the MTAG method, researchers identified a new locus near the CD28gene, specific to East Asian populations, as well as two new pleiotropic regions at 9q32 and 10q25.2^81^, ^82^. Furthermore, a candidate gene study confirmed that variations in CLEC16Aare associated with a reduced risk of AR, playing a protective role^83^.

The genetic architecture of AR highly overlaps with that of other allergic diseases. Studies have confirmed a strong genetic correlation between AR and asthma and eczema, with over 100 shared risk genes identified^78^. At the level of the "allergic march," GWAS has identified genetic loci, such as rs9565267, associated with the specific trajectory of progression from atopic dermatitis (AD) to AR^84^. Research has also genetically confirmed that AR is a causal risk factor for food allergy, with the connection established through shared loci in the HLA region^85^.

However, the effects of some genetic loci are specific. For example, polymorphisms in the GSDMB gene are more strongly associated with asthma and asthma with comorbid AR than with AR alone, revealing the complexity of the genetic background of allergic diseases^79^.

### Variations in Antigen Presentation (HLA) and Epithelial Barrier (FLG) Genes

The genetic susceptibility to allergic rhinitis (AR) involves multiple genes, among which variations in the human leukocyte antigen (HLA) and filaggrin (FLG) genes are two of the most extensively studied risk factors. They represent the immune system's "internal recognition" of allergens and the epithelial tissue's "external defense," respectively, and are central to AR genetic research.

### Antigen Presentation (HLA) Gene Variations

The human leukocyte antigen (HLA) system is responsible for presenting antigen peptides in the adaptive immune response, and its genetic variations are one of the most significant genetic risk factors for AR. A large-scale GWAS meta-analysis of over 210,000 individuals of European ancestry identified the HLA region as one of the strongest susceptibility loci for AR^86^. Through fine-mapping analysis, the study further found that the risk signals were directly related to specific amino acid variations in the peptide-binding pockets of HLA-DQB1 and HLA-B molecules, suggesting that genetic differences may influence AR susceptibility by altering the efficiency of allergen presentation^86^.

This association in the HLA region has also been confirmed in different ethnic groups. In a replication study in the Han Chinese population, SNP rs7775228 in the HLA region was confirmed to be significantly associated with AR risk, and its risk allele was linked to elevated serum total IgE levels^87^. The genetic architecture of AR is complex, and research has also begun to explore the interaction between HLA and other genes (such as TYRO3) to fully understand its pathogenic mechanisms^88^.

### Epithelial Barrier (FLG) Gene Variations

Filaggrin (FLG) is a key protein for maintaining epithelial barrier function, and its loss-of-function (LoF) mutations are the strongest genetic risk factor for atopic dermatitis (AD)^89^. By compromising barrier integrity, FLG mutations play an upstream driving role in the "Atopic March," significantly increasing the subsequent risk of developing AR and asthma. A path analysis of a longitudinal birth cohort study showed that FLG mutations primarily lead to AR and asthma by causing early-onset eczema, which in turn promotes sensitization to aeroallergens^90^. At the same time, the study also found that FLG mutations might have a direct effect on rhinitis at age 10, independent of eczema, suggesting a possible role in the nasal mucosal barrier as well^90^.

The frequency and types of FLG mutations show significant heterogeneity across global populations. For example, common FLG-LoF mutations in European populations have a similar carrier rate in the Chilean population^91^, but are extremely rare and not significantly associated with the disease in Turkish children^92^. Additionally, specific types of FLG mutations have been found in African American and Saudi populations, and these mutations are all associated with allergic diseases such as AR^89^, ^93^.

Genetic risk and environmental factors jointly influence disease occurrence. One study compared two major risk factors for allergic diseases like atopic dermatitis and asthma. It found that the risk to offspring from a poor maternal diet during pregnancy was comparable to the genetic risk of carrying FLG mutations. This highlights that impaired epithelial barrier function, whether due to congenital genetic factors or acquired environmental factors, is a key link in the pathogenesis of allergic diseases^94^.

## Variations in Cytokine and Alarmin Signaling (IL4R, TSLP, etc.) Genes

After focusing on the macroscopic picture revealed by GWAS and the two key genes, HLA and FLG, we delve deeper into the core molecular pathways driving the allergic reaction. The pathogenesis of allergic rhinitis is closely related to genetic variations in Th2 cytokine and epithelial alarmin signaling pathways^95^-^98^. These variations, by affecting the expression and function of signaling molecules, modulate an individual's immune response intensity to allergens, thereby determining the genetic susceptibility to AR^96^, ^97^.

### Thymic Stromal Lymphopoietin (TSLP) Signaling Pathway

As a key epithelial alarmin, genetic variations in TSLP are an important risk factor for AR^98^, ^99^. For example, the polymorphism rs3806933 in the TSLP promoter region is significantly associated with the risk of allergic rhinitis in the Korean population and is considered a key genetic risk driving the "atopic march"^98^. Another study found that the risk allele [C] of TSLP SNP rs2289277 significantly increased the likelihood of having multiple comorbid allergic diseases^99^. Furthermore, the upstream Toll-like receptor 3 (TLR3) signaling pathway can induce TSLP expression, and variations in the TLR3 locus are directly associated with sensitization to aeroallergens, revealing a genetic link between innate immunity and alarmin signaling^100^.

### Interleukin-4 Receptor (IL4R) and Downstream Signaling Pathways

IL-4 and IL-13 are the core cytokines driving AR inflammation, and they primarily activate the downstream STAT6 signaling pathway through the IL-4 receptor alpha chain (IL4Rα)^96^. Therefore, genetic variations in IL4R have a significant impact on AR susceptibility. Studies have confirmed that the variant genotypes of the functional missense mutations I50V (rs1805010) and Q576R (rs1801275) in the IL4R gene are both significantly associated with the risk of atopy^97^. These genetic variations may also play a role in a broader range of immune-related diseases; for instance, a study exploring the relationship between allergies and childhood leukemia also analyzed the interaction between IL4R-rs1801275 and a history of allergies^101^.

The complexity of genetic susceptibility is reflected in gene-gene interactions: one study found that loss-of-function mutations in the skin barrier gene FLG only significantly increased the risk of allergic sensitization in individuals homozygous for the IL4R SNP rs3024676, suggesting a synergistic pathogenic effect between immune dysregulation and barrier function defects^102^. Downstream in this signaling pathway, the TT homozygous genotype of the STAT6 SNP rs324011 has also been confirmed to be significantly associated with the risk of allergic asthma and elevated serum total IgE levels, further completing the genetic picture of this pathway^96^. Furthermore, STAT6 gain-of-function mutations have been identified in humans and are associated with allergic disease, further highlighting the centrality of this pathway in immune dysregulation.

### Interleukin-33 (IL-33) Signaling Pathway

IL-33 is another important epithelial-derived alarmin that also plays a significant role in the pathogenesis of AR. Large-scale exome sequencing analysis shows that among genes associated with asthma and allergy, the region containing IL33 and its receptor IL1RL1 is one of the gene clusters with the strongest signals, with numerous rare variants significantly affecting disease risk^95^. A prospective cohort study of Finnish children found that the variant genotype of the IL33 gene SNP rs1342326 was significantly associated with the risk of allergic rhinitis in school-aged children, with a corrected odds ratio (OR) as high as 3.23^103^. At the molecular level, another study revealed that the risk "T" allele of the intronic SNP rs4742170 in IL33 could impair the binding site of the glucocorticoid receptor (GR), thereby weakening its inhibitory effect on IL33 expression, which may lead to exacerbated inflammation^104^. Experimental animal model studies also indicate that although the functions of TSLP and IL-33 do not completely overlap, both play a role in allergic inflammation, with TSLP being an important upstream regulator of IL-13^105^.

### Other Related Signaling Pathways

The high-affinity IgE receptor (FcεRI) is the core effector molecule mediating allergic reactions^106^. Research has found that the SNP rs36233990 in the promoter region of the FCER1G gene, which encodes its gamma chain, is significantly enriched in patients with allergic rhinitis and comorbid asthma, directly linking genetic variation to the core effector phase of AR^106^.

In summary, genetic variations in cytokine and alarmin signaling pathways constitute a complex network of susceptibility to allergic rhinitis. Evidence from large-scale genomic and candidate gene studies collectively points to three key signaling pathways: TSLP, IL-4R, and IL-33. Functional SNPs in these pathways, whether common or rare, can set the threshold for an individual's immune response by affecting ligand expression, receptor function, or downstream signal transduction. Furthermore, gene-gene interactions further reveal the complexity of the genetic background, suggesting that an integrated perspective is needed to fully understand the genetic architecture of allergic rhinitis.

To clearly present the numerous susceptibility genes, loci, and their specific functions related to allergic rhinitis mentioned in the preceding sections, we have summarized this key information by category in Table 4 for systematic reference and comparison.

## Epigenetic Regulation at the Gene-Environment Interface

Allergic rhinitis (AR) is the product of complex interactions between genetics and the environment, and its rapidly increasing prevalence highlights the importance of environmental factors^107^, ^108^. Epigenetics, particularly DNA methylation, serves as a key bridge connecting genes and the environment; it can translate the effects of environmental factors (such as pollutants, maternal status) into stable gene expression regulation without altering the DNA sequence, thereby influencing susceptibility to AR^107^.

**Figure 4:**
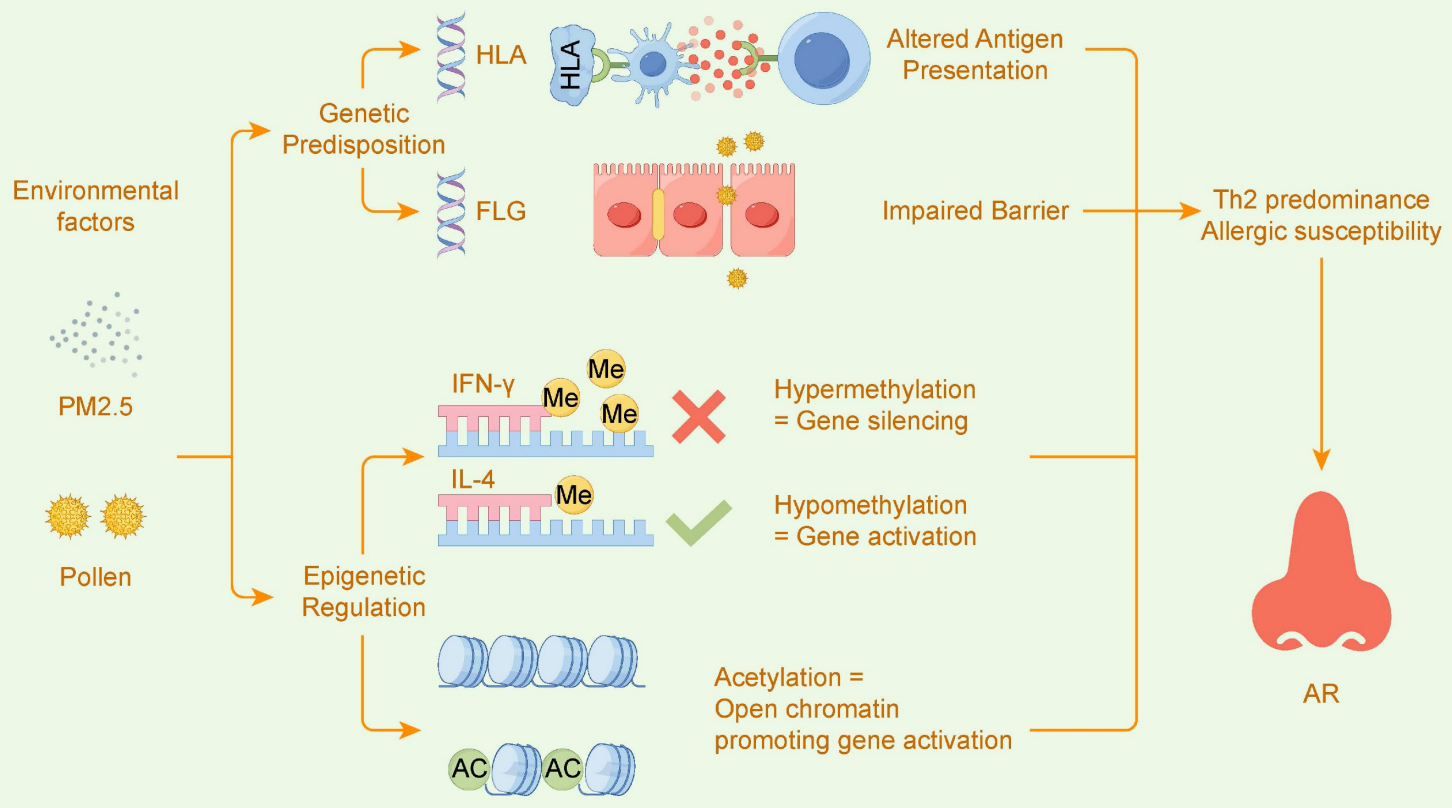
Gene-Environment Interactions Drive AR via Epigenetic Regulation. This figure illustrates how allergic rhinitis (AR) is driven by the interplay of Genetic Predisposition and Environmental factors, mediated by Epigenetic Regulation. Genetic variants in genes like HLA and FLG can lead to Altered Antigen Presentation and an Impaired Barrier. Environmental exposures, such as to PM2.5 and pollen, induce epigenetic changes. These changes include Hypermethylation which leads to the silencing of Th1-related genes like IFN-Y, and Hypomethylation or histone Acetylation, which promotes the activation of Th2-related genes like IL-4. This epigenetic imbalance results in Th2 predominance and Allergic susceptibility, ultimately leading to AR.

Figure 4 intuitively illustrates this core mechanism: how genetic predisposition (e.g., variations in HLA and FLG genes) and environmental factors (e.g., exposure to PM2.5 and pollen) converge through the intermediary of epigenetic regulation (e.g., DNA methylation and histone acetylation) to disrupt immune balance, ultimately leading to the development of allergic rhinitis.

### Regulation of Th1/Th2-Related Genes by DNA Methylation

The core pathological feature of AR is the shift of the Th1/Th2 immune balance towards a Th2 phenotype, and DNA methylation is a key switch regulating this balance^109^. Environmental factors, such as PM2.5 exposure or season of birth, can induce hypermethylation of the promoter of the key Th1 cytokine IFN-γ gene in CD4+ T cells through upstream signaling pathways (e.g., ERK-DNMT)^110^. This hypermethylation suppresses the transcription of IFN-γ, weakens the Th1 immune response, and thus promotes a Th2-dominant state conducive to allergies^110^. Corresponding to the suppression of Th1 genes, large-scale population studies have found that in children's nasal samples, the promoter regions of key Th2 genes associated with allergic inflammation (such as IL-4 and IL-13) exhibit a state of hypomethylation, which facilitates their expression and the activation of the Th2 response^111^.

This regulatory network also involves key transcription factors. For instance, Foxp3, the key transcription factor for regulatory T cells (Tregs), is also regulated by methylation. Hypermethylation of its promoter is associated with the suppression of Treg function, and this methylation pattern may be influenced by maternal AR status, suggesting the potential for intergenerational transmission of epigenetic imprints^112^, ^113^. Recent studies have further revealed the upstream molecular pathways that regulate this process, with DNA methyltransferases (DNMTs) playing a central role. For example, DNMT1 is significantly upregulated in AR models and drives Th2 cell differentiation by promoting the methylation of the FOXO3 gene promoter to inhibit its expression, which in turn activates the downstream NF-κB/GATA3 signaling pathway^109^. Additionally, the transcription factor FOXC1 can also mediate the methylation of other genes by activating DNMT3B, collectively participating in the complex epigenetic regulatory network^114^.

### Histone Modification and Gene Activation

In addition to DNA methylation, histone modification is another critical layer in the gene-environment interaction that regulates gene expression. Activating histone modifications, such as the acetylation of histone H3 at lysines 9 and 14 (H3K9/14Ac) and the trimethylation of lysine 4 (H3K4me3), are important marks of active gene transcription^115^, ^116^. These modifications can establish a "permissive" chromatin structure, enhancing the accessibility of DNA and creating the prerequisite for gene activation^117^, ^118^.

External environmental signals (such as cytokines or antigen stimulation) activate key transcription factors, like GATA-3, STAT6, and NF-κB, through intracellular signaling pathways^119^, ^120^. These transcription factors then target the regulatory elements (such as promoters and enhancers) of specific genes and recruit histone-modifying enzymes and chromatin remodeling complexes. For example, in Th2 cells, the c-Myb and GATA-3 complex can recruit the histone methyltransferase MLL to mediate H3K4 methylation and H3K9 acetylation at the Il13 locus, thereby robustly activating its expression^121^. Similarly, in B cells, the allergy-associated protein PHF11 also enhances IgE class switching by promoting permissive histone modifications in the GLTε promoter region^115^.

Specific DNA regulatory elements play a platform role in this process. The HS2 enhancer of the Il4 gene is a key target for GATA-3, which coordinates activating histone modifications across the entire locus by binding to HS2; in the absence of HS2, even the overexpression of GATA-3 cannot activate IL-4^122^. Furthermore, different subtypes of chromatin remodeling enzymes also have regulatory specificity. For example, the inhibitory subunit BAF180 of the SWI/SNF complex suppresses the Il10 gene in normal Th2 cells. When it is absent, the activating subunit BAF250 can bind, leading to increased levels of histone acetylation and gene activation^123^.

Gene activation involves not only "turning on" but also the removal of inhibitory mechanisms. In Th1 cells, the normal function of the STAT1 signaling pathway is to inhibit the formation of activating histone modifications at the Il4 locus^117^; in Th2 cells, the Th2 LCR (locus control region) is responsible for suppressing the epigenetic activation of the ifng locus^124^. Defects in these regulatory pathways can lead to the modification of incorrect genes with activating histone marks, thereby triggering an abnormal immune response. It is noteworthy that "environmental" stimuli are not limited to biomolecules. Physical factors such as low-level laser therapy (LLLT) can also enhance the expression of pro-inflammatory cytokines like TNF-α by inducing activating histone modifications (H3/H4 acetylation and H3K4 trimethylation) on their genes^116^. This suggests that cells can translate various forms of external stimuli into a common epigenetic language to regulate gene activation.

### Post-transcriptional Regulation by MicroRNAs (miRNAs)

Beyond transcriptional regulation, such as DNA methylation and histone modification, gene expression is also subject to precise post-transcriptional control, in which microRNAs (miRNAs) are key molecules that regulate by degrading target gene mRNA or inhibiting its translation^125^. In allergic rhinitis (AR), miRNAs represent another important class of epigenetic regulators connecting environmental exposure to disease development. Due to their high stability in bodily fluids like plasma, miRNAs also show great potential as non-invasive biomarkers for disease diagnosis and severity assessment^126^. Exposure to environmental allergens is a core driver of changes in miRNA expression profiles. For example, seasonal AR patients show significant differences in their miRNA expression profiles during versus outside the allergy season^127^, The miRNA-mRNA regulatory network within their CD4+ T cells also changes accordingly, clearly reflecting the gene-environment interaction^128^.

In the pathophysiology of AR, the functions of miRNAs are diverse and complex. On the one hand, many miRNAs exhibit pro-inflammatory effects and are upregulated in AR patients. For example, miR-223-3p promotes eosinophil activation and inflammation by targeting FBXW7 and INPP4A^125^, ^129^; miR-126-5p activates the NF-κB inflammatory pathway by targeting HIPK2^130^; and miR-29a-3p disrupts the integrity of the nasal epithelial barrier by targeting the CTNNB1-VCL module^131^. On the other hand, some miRNAs play an important immunosuppressive role, such as miR-146a, which is downregulated in AR patients and can enhance the differentiation and function of regulatory T cells (Tregs) by positively regulating its target gene STAT5b^132^.

The regulatory role of miRNAs is not limited to a single "miRNA-target gene" axis but exists within a more complex regulatory network. Long non-coding RNAs (lncRNAs) can act as "miRNA sponges," regulating miRNA activity through competitive binding (the ceRNA mechanism). For example, in AR, the lncRNAs HCP5, NEAT1, and GAS5 are involved in regulating Treg function and the Th1/Th2 immune balance by sponging miR-16, miR-21/miR-125a, and miR-21/miR-140, respectively^133^-^135^. Furthermore, this regulation can also be systemic; extracellular vesicles (EVs) in the plasma of AR patients can carry functional miR-150-5p and deliver it to distant immune cells, thereby promoting the Th2/ILC2 response^136^.

The interaction between genes and the environment leaves its mark even at the beginning of life. Studies have found that specific miRNA expression profiles in neonatal cord blood (such as miR-149-5p, miR-125a-5p) are significantly associated with the risk of developing childhood AR, indicating that early-life epigenetic features may predict future disease susceptibility^126^.

In summary, from DNA methylation and histone modifications to post-transcriptional regulation by miRNAs, these epigenetic mechanisms collectively form the core network connecting environmental stimuli to gene expression. Through multi-layered and networked collaboration, they are deeply involved in and drive the pathological process of allergic rhinitis, opening new avenues for the early diagnosis, risk assessment, and targeted treatment of the disease.

## The Influence of the Microbiome: Dysbiosis and the Gut-Nose Axis

### Nasal Microbiota Dysbiosis

Allergic Rhinitis (AR) is closely associated with the dysbiosis of the nasal microbial community, a condition reflected in multiple aspects including community composition, diversity, structure, and function^137^, ^138^. The nasal microecological balance in AR patients is disrupted, showing significant differences from that of healthy individuals.

In terms of composition, the nasal cavity of AR patients shows a significant increase in the abundance of Firmicutes and Bacteroidota, whereas the relative abundance of Actinobacteriota is significantly reduced^137^, ^139^, ^140^. At the genus level, this dysbiosis is more specific, marked by the significant enrichment of *Staphylococcus*, especially *S. aureus* 139, 141. Concurrently, the abundance of genera such as *Dolosigranulum*, *Haemophilus*, and *Moraxella* is also significantly elevated^137^. Conversely, some genera common in healthy individuals, such as *Ralstonia* and *Corynebacterium*, are significantly reduced^141^, ^142^, thus forming a unique disease-associated microbial signature^138^.

Regarding changes in microbial diversity, although conclusions on alpha diversity (community richness and evenness) vary across studies^137^, ^140^, ^143^, the results for beta diversity (differences in community structure) are highly consistent. Multiple studies have confirmed that the overall structure of the nasal microbiota in AR patients differs significantly from that of healthy control groups^137^, ^138^, ^144^. This structural shift is accompanied by a more complex network of microbial interactions, suggesting instability in the nasal ecosystem of AR patients^137^.

Changes in microbial composition also lead to functional shifts. Functional prediction analysis shows that the nasal microbiota of AR patients exhibits differential expression across multiple metabolic pathways, mainly involving degradation and biosynthesis processes^137^. For example, functions like the pentose phosphate pathway are enriched, while functions such as primary bile acid biosynthesis are diminished. These changes may modulate the host's local immune response by affecting metabolic products, thereby exacerbating the inflammatory process of AR^140^.

### The Gut-Nose Axis and Long-Range Immunomodulation

While exploring the local nasal microenvironment, the emerging concept of the "gut-nose axis," derived from the "gut-lung axis" theory, reveals that the gut microbiota can influence the course of allergic rhinitis (AR) through long-range immunomodulation, offering new avenues for treatment^145^-^147^.

Gut microbiota dysbiosis is a key factor in the pathogenesis of AR. It alters the body's immune-inflammatory state by affecting the differentiation of systemic immune cells, thereby increasing susceptibility to AR^145^, ^148^. Fecal microbiota transplantation (FMT) experiments have confirmed this causal relationship: transplanting the fecal microbiota from AR mice to healthy mice can induce AR-like symptoms in the recipients^145^.

**Figure 5:**
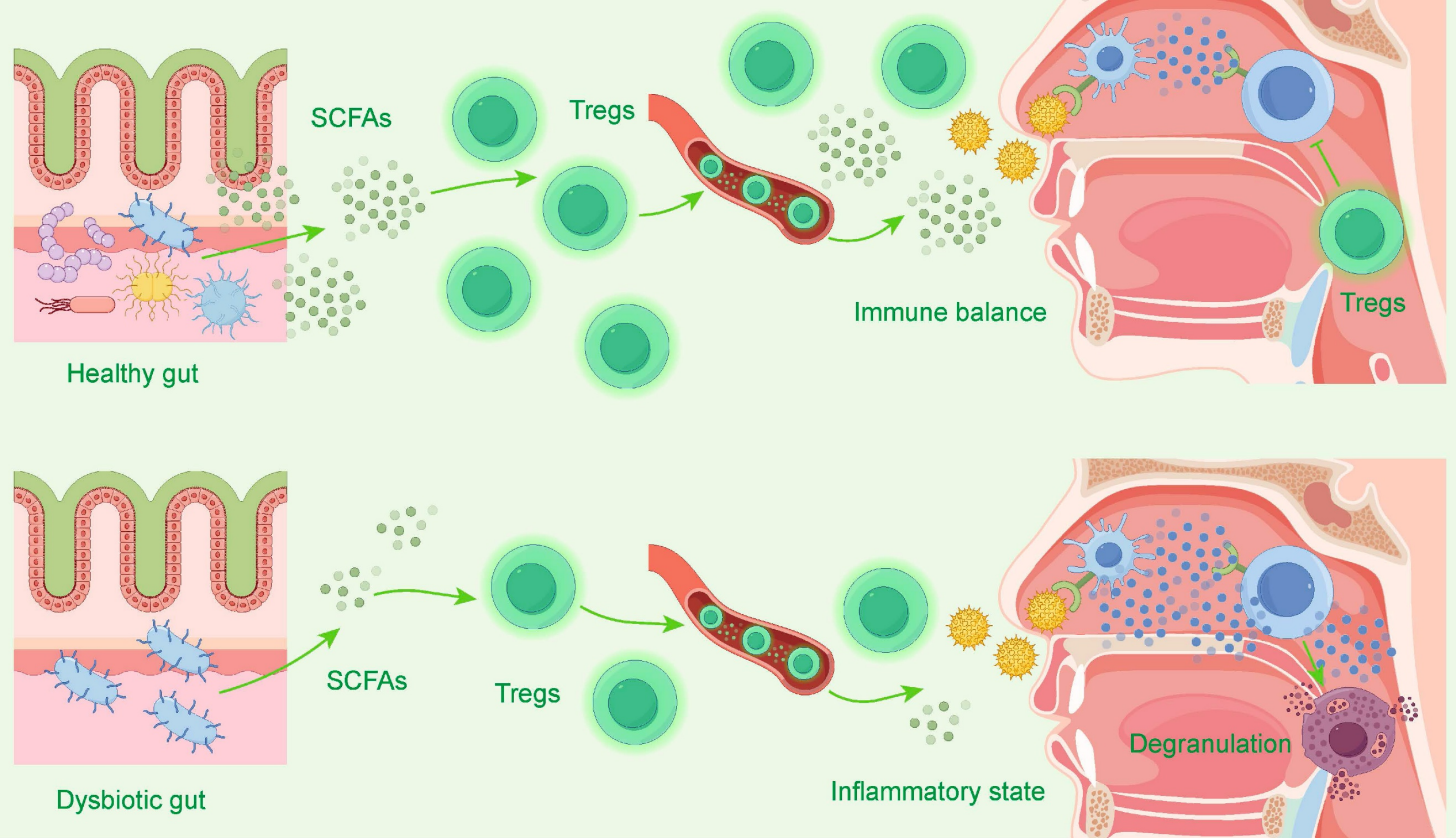
The Gut-Nose Axis: Microbiota's Regulation of Nasal Immunity. This diagram illustrates the "gut-nose axis." In the top panel, a Healthy gut produces abundant short-chain fatty acids (SCFAs), which promote regulatory T cells (Tregs). These Tregs travel to the nasal cavity to maintain Immune balance. In the bottom panel, a Dysbiotic gut produces fewer SCFAs, leading to insufficient Tregs. This lack of regulation results in a nasal Inflammatory state, characterized by mast cell Degranulation and allergic inflammation.

This cross-organ regulation views the human body as a unified immune entity^149^. Metabolites produced by the gut microbiota, such as short-chain fatty acids (SCFAs), are key signaling molecules that can regulate the Th17/Treg cell balance and maintain immune homeostasis^147^, ^148^. Figure 5 visually demonstrates this core regulatory mechanism of the "gut-nose axis": a healthy gut microbiota promotes the differentiation and function of regulatory T cells (Tregs) by producing metabolites such as short-chain fatty acids (SCFAs). These Tregs then migrate to the nasal cavity to maintain local immune balance. Conversely, when the gut microbiota is dysbiotic, SCFA production decreases and Treg function is impaired, leading to nasal immune imbalance and exacerbated inflammation. Studies have found that the exacerbation of AR symptoms is associated with a decrease in SCFA-producing bacteria (such as *Ruminococcus*) and a decline in SCFA levels. Conversely, direct supplementation with sodium butyrate can alleviate symptoms by upregulating Treg cell-related pathways^147^.

In summary, the gut-nose axis theory elucidates how gut microbiota imbalance affects AR through metabolites and the systemic immune system. Interventions targeting the gut microbiota, such as FMT, probiotics, and traditional Chinese medicine, can indirectly act on the nasal mucosal immune system, providing promising new strategies for the treatment of AR^145^, ^149^.

## A Translational Medicine Perspective: From Pathophysiology to Targeted Therapy

A deep understanding of the pathophysiological mechanisms of allergic diseases, especially allergic rhinitis (AR) and its related comorbidities, has greatly driven the innovation of therapeutic strategies. The development of translational medicine has enabled a shift from traditional symptomatic treatments to precision interventions targeting the core molecular pathways of diseases. This chapter will focus on three landmark biologic agents—omalizumab (targeting IgE), dupilumab (targeting IL-4/IL-13), and tezepelumab (targeting TSLP)—and explain how they have successfully translated basic pathophysiological knowledge into highly effective clinical treatment plans.

**Figure 6:**
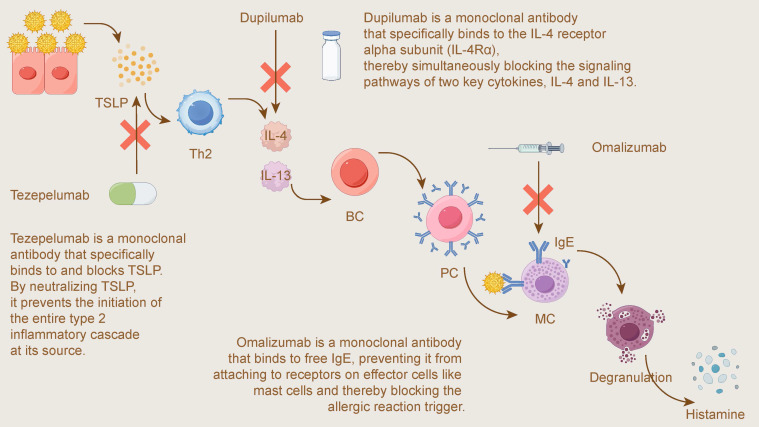
Mechanisms of Action for Biologics Targeting the Type 2 Inflammatory Pathway. This figure illustrates the mechanisms of three key biologics targeting different stages of the type 2 inflammatory cascade. Tezepelumab is a monoclonal antibody that acts upstream by binding to and blocking the alarmin TSLP, preventing the initiation of the inflammatory cascade at its source. Dupilumab is a monoclonal antibody that binds to the IL-4 receptor alpha subunit (IL-4Rα), thereby simultaneously blocking the signaling pathways of two key Th2 cytokines, IL-4 and IL-13. Omalizumab is a monoclonal antibody that targets the final effector molecule by binding to free IgE, preventing it from attaching to receptors on effector cells like mast cells (MC) and thereby blocking the trigger for Degranulation and Histamine release.

Figure 6 visually illustrates the different targets and mechanisms of these three biologics within the type 2 inflammatory pathway: from the most upstream "alarmin" TSLP, to the key midstream cytokines IL-4/IL-13, and finally to the downstream effector molecule IgE, together forming a multi-level precision intervention strategy.

### Omalizumab (Targeting IgE)

Allergic rhinitis (AR) is a classic IgE-mediated type I hypersensitivity disease, in which IgE plays a central role in triggering the release of inflammatory mediators from mast cells and basophils^150^, ^151^. Based on this key pathophysiological mechanism, therapies targeting IgE were developed. Omalizumab is a recombinant humanized monoclonal antibody that binds to free IgE in the serum, preventing it from binding to the high-affinity receptor (FcεRI) on the surface of effector cells. Thereby effectively interrupting the upstream allergic cascade and downregulating FcεRI expression^150^-^152^.

The clinical efficacy of omalizumab has been confirmed in multiple high-level studies. In a Phase III randomized controlled trial (RCT) involving patients with severe pollinosis who responded poorly to standard-of-care (SoC), the addition of omalizumab significantly improved nasal and ocular symptoms, quality of life (QoL), and work productivity^152^. Real-world studies also support its efficacy in both seasonal AR (SAR) and perennial AR (PAR), with particularly significant improvement in nasal congestion symptoms, and demonstrate that long-term treatment (36 months) is safe and effective^153^-^155^.

The practice of translational medicine involves not only drug development but also the optimization of treatment strategies. Given the cyclical nature of SAR, pre-seasonal prophylactic treatment has become an innovative application. Both RCTs and retrospective studies have confirmed that a single injection of omalizumab (150mg or 300mg) before the pollen season can effectively control symptoms throughout the season and reduce the use of other medications^156^, ^157^.

To achieve personalized precision medicine, researchers are actively exploring predictive indicators of efficacy. One study found that the ratio of total IgE levels at 16 weeks of treatment to baseline (≥2.0) can serve as a biomarker for predicting clinical response^158^. Additionally, patients with more severe baseline symptoms often experience greater improvement from the treatment^153^.

The translational applications of omalizumab are extensive. It not only translates clinical benefits into socioeconomic value by significantly reducing work productivity loss due to severe pollinosis^159^, but has also shown efficacy in other IgE-mediated diseases such as chronic urticaria^160^. Particularly noteworthy is a case report showing that after omalizumab effectively controlled severe AR in a child with autism spectrum disorder (ASD), the child's behavioral symptoms also improved, opening new avenues for exploring the "immune-brain" axis interaction^161^.

In summary, omalizumab is a prime example of successful translational medicine. It has transformed a deep understanding of AR pathophysiology into an effective and continually optimized targeted therapy, providing an important treatment option for patients with refractory AR.

Following the success of omalizumab in targeting the final effector molecule of the allergic cascade, IgE, researchers have turned their attention further upstream to the key cytokines that drive type 2 inflammation, opening up new therapeutic pathways.

### Dupilumab (Targeting IL-4/IL-13)

In the pathophysiology of type 2 inflammatory diseases such as allergic rhinitis (AR), interleukin-4 (IL-4) and interleukin-13 (IL-13) are key driving factors^162^. They signal through a shared IL-4 receptor alpha subunit (IL-4Rα), inducing IgE production, recruiting inflammatory cells, and compromising epithelial barrier integrity^162^, ^163^. The understanding of this core mechanism directly spurred the development of dupilumab, a precision-targeted therapeutic drug.

Dupilumab is a fully human monoclonal antibody that specifically binds to IL-4Rα, simultaneously blocking the signaling pathways of both IL-4 and IL-13^162^, ^164^. Its efficacy is not only reflected in clinical symptom improvement but also extends to the molecular level. A transcriptomic study confirmed that dupilumab can significantly "normalize" the disease-associated gene expression profile in the nasal tissue of AR patients (NES = -1.73, p = .002), whereas allergen-specific immunotherapy (SCIT) alone has no such effect^163^. Furthermore, clinical studies have consistently shown that dupilumab significantly reduces the levels of type 2 inflammatory biomarkers such as serum total IgE, TARC, and eosinophils^165^-^167^.

As AR often coexists with other type 2 inflammatory diseases, dupilumab, as a systemic therapy, demonstrates unique translational medicine value in treating these comorbidities:

- **Asthma and AR:** Post-hoc analyses show that while effectively controlling moderate-to-severe asthma, dupilumab also significantly improves nasal symptoms and related quality of life in patients with comorbid perennial allergic rhinitis (PAR)^164^, ^165^.
- **CRSwNP and AR:** In an analysis of large Phase 3 clinical trials, dupilumab showed significant efficacy in treating chronic rhinosinusitis with nasal polyps (CRSwNP), and this efficacy was not affected by whether the patients had comorbid AR^166^.
- **AD and AR:** Dupilumab is currently the only asthma biologic proven effective for atopic dermatitis (AD)^168^. In a Phase 3 clinical trial in adolescent AD patients, its efficacy far surpassed that of a placebo^169^. Real-world studies confirm that it can simultaneously improve the symptoms of both AD and comorbid AR^167^, and that comorbid AR is a positive predictor for a rapid response in AD patients^170^, ^171^.

In addition to its use as a monotherapy, dupilumab has shown great potential in cutting-edge combination therapy strategies. A Phase 2a clinical trial indicated that when dupilumab was combined with SCIT for AR, it significantly improved the tolerability of SCIT, allowing more patients to reach the maintenance dose and reducing the use of epinephrine^172^. Similarly, in severe, refractory AD, combining it with SCIT not only led to sustained clinical improvement but also induced favorable immunological changes, such as an increase in specific IgG4^173^.

In conclusion, dupilumab is an excellent example of translational medicine. By precisely targeting the core pathophysiological pathways of AR and related comorbidities, it not only provides a highly effective treatment option for patients with type 2 inflammatory diseases but also opens new paths for optimizing existing therapies and tackling refractory conditions.

While targeting key Type 2 cytokines like IL-4 and IL-13 has proven to be an effective strategy, more advanced translational medicine research is exploring the possibility of blocking the "alarmins" located at the very top of the inflammatory cascade, aiming to regulate a broader range of inflammatory pathways.

### Tezepelumab (Targeting the Upstream Alarmin TSLP)

Thymic stromal lymphopoietin (TSLP) is a key epithelial-derived "alarmin" situated upstream in the inflammatory cascade, capable of driving the Type 2 inflammatory response^174^, ^175^. As a cytokine released in response to various stimuli such as allergens, microbes, and physical injury, TSLP can activate multiple immune cells, including type 2 innate lymphoid cells (ILC2s), mast cells, and T helper 2 (Th2) cells^175^, ^176^. Tezepelumab is a humanized monoclonal antibody that specifically binds to and blocks TSLP, thereby broadly inhibiting downstream inflammatory pathways^174^, ^177^.

The pivotal Phase III NAVIGATOR study and its long-term extension study, DESTINATION, confirmed that tezepelumab can significantly and continuously reduce the annualized asthma exacerbation rate (AAER) in patients with severe asthma, improve lung function and quality of life. Its efficacy is not limited by baseline Type 2 biomarker levels, and it has shown a good long-term safety profile^174^, ^177^.

Tezepelumab is also effective in several challenging patient subgroups. In patients with chronic rhinosinusitis with nasal polyps (CRSwNP), it not only reduces the AAER by 85% but also significantly improves nasal symptoms (SNOT-22 score)^174^, ^178^. For patients with persistent airflow obstruction (PAO), tezepelumab can also effectively reduce the AAER and improve lung function^179^. Furthermore, in patients with aspirin or non-steroidal anti-inflammatory drug (NSAID) sensitivity, its effect on reducing the AAER is particularly pronounced, with a reduction of 83%^180^. Its efficacy in the Japanese patient population is also consistent with global data^181^.

Translational medicine research has further revealed its potential. In a combination therapy trial, tezepelumab enhanced the efficacy of allergen subcutaneous immunotherapy (SCIT) and showed some sustained clinical benefits even one year after discontinuation, indicating a potential to induce long-term immune tolerance^182^, ^183^. This effect is associated with the sustained downregulation of Type 2 inflammation-related genes in the nasal mucosa, particularly tryptase (TPSAB1)^182^.

In conclusion, tezepelumab, by targeting the upstream alarmin TSLP, provides a broad-spectrum and effective new therapeutic strategy for severe asthma and related allergic diseases. Its mechanism of action allows it to cover a wider range of patients and brings new hope for achieving long-term disease remission.

To systematically summarize and compare the key information of the three targeted biologics discussed above, Table 5 provides a multi-dimensional summary of their targets, mechanisms of action, and key clinical evidence and applications.

### Conclusion and Future Perspectives

**Figure 7:**
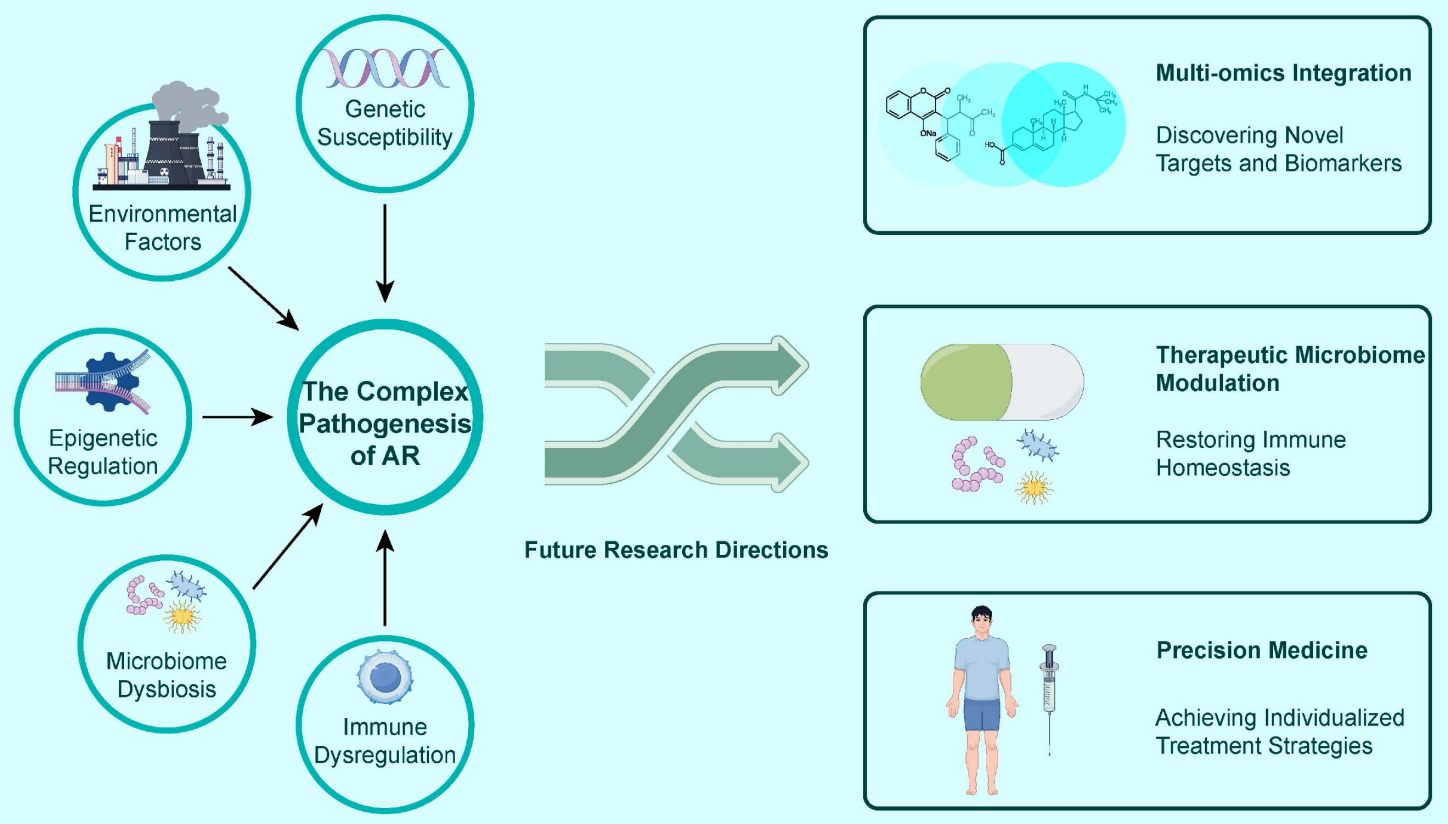
The Complex Pathogenesis of AR and Future Research Directions. This figure summarizes that The Complex Pathogenesis of AR is driven by an interplay of factors including Genetic Susceptibility, Environmental Factors, Epigenetic Regulation, Microbiome Dysbiosis, and Immune Dysregulation. Key Future Research Directions include: Multi-omics Integration for Discovering Novel Targets and Biomarkers; Therapeutic Microbiome Modulation aimed at Restoring Immune Homeostasis; and Precision Medicine for Achieving Individualized Treatment Strategies.

The core concepts of this chapter are summarized in Figure 7, which systematically outlines the complex, multifactorial pathogenesis of allergic rhinitis and highlights the three most promising future research directions, serving as a condensed summary and forward-looking conclusion for the entire review.

## Conclusion

The pathogenesis of allergic rhinitis is a complex biological process involving the synergistic action of multiple layers, links, and targets, rather than a single linear pathway. This review has systematically delineated this panoramic view: from the rapid initiation by innate immunity (epithelial cells-alarmins-ILC2s) to the amplification and maintenance by adaptive immunity (Th2-cytokines-IgE-effector cells); from the sophisticated signaling networks regulating these cellular behaviors (the IL-4/STAT6/GATA3 axis) to the genetic background determining individual susceptibility (variations in genes like HLA, FLG); from the epigenetic modifications connecting the environment and genes (DNA methylation, miRNAs) to the microbiome affecting local and distant immune homeostasis (nasal microbiota dysbiosis and the gut-nose axis). This series of interconnected links collectively constitutes the immunogenetic basis of AR. It is precisely based on a profound understanding of these core pathophysiological links that translational medicine has achieved major breakthroughs, leading to the development of targeted therapeutic drugs such as omalizumab, dupilumab, and tezepelumab, which can precisely block key nodes and have brought revolutionary changes to clinical practice.

### Future Perspectives

Although we have made considerable progress, a complete cure for AR still faces challenges. Future research should focus on the following cutting-edge directions:

**Multi-omics Integrated Analysis and Disease Endotyping:** Future research needs to move beyond single-dimensional analysis by integrating multi-omics data, including genomics, transcriptomics, proteomics, metabolomics, and microbiomics, to construct a systems biology model of AR. This will help us identify new pathogenic pathways and biomarkers and, more importantly, enable "endotyping" based on patients' unique molecular characteristics. This will allow for the differentiation of disease subtypes driven by different molecular pathways (e.g., TSLP-driven, IL-33-driven), laying the foundation for achieving true personalized medicine.

**Therapeutic Modulation of the Microbiome:** The "gut-nose axis" theory provides a new perspective for the prevention and treatment of AR. Future research priorities should include: further elucidating the specific molecular mechanisms by which the gut microbiota and its metabolites (such as short-chain fatty acids) regulate nasal immunity; developing new intervention strategies based on modulating the microbiome, such as designing more targeted probiotics, prebiotics, or postbiotics; and evaluating the long-term efficacy and safety of fecal microbiota transplantation (FMT) in reshaping immune homeostasis and treating refractory AR.

**Developing More Precise Personalized Medical Strategies:** Based on disease endotyping, future treatments will become more precise. For example, by detecting a patient's biomarker profile, it may be possible to predict their responsiveness to specific biologics (such as anti-IL-4Rα or anti-TSLP), thereby "choosing the right drug for the right patient." In addition, exploring the combined use of biologics and allergen immunotherapy (AIT) with the aim of inducing deeper and more lasting immune tolerance will be an important direction for achieving disease remission or even a functional cure.

In conclusion, with the continuous development of multi-omics technologies and our deepening understanding of the immunogenetic network of AR, the future management of AR will gradually transition from the current symptomatic control to a new era of precision prevention, diagnosis, and targeted therapy based on individual molecular characteristics.

## Figures and Tables

**Table 1:** Role of Th2 Cells and Their Key Cytokines in Allergic Rhinitis

Immune Component	Main Function	Key Mechanisms	Reference
Th2 Cell	Core of the type 2 immune response; initiates the allergic cascade.	Allergens presented by APCs to naive CD4+ T cells, which differentiate into Th2 cells under cytokines like IL-4; Differentiation is dominated by the key transcription factor GATA3, regulated by the IL-4/JAK/STAT6 signaling pathway; Increased number of Th2 cells in AR patients leads to a Th1/Th2 imbalance skewed towards Th2.	1-3, 21
IL-4	Drives Th2 cell differentiation and induces B cells to produce allergen-specific IgE antibodies.	Drives naive T cell differentiation towards Th2 phenotype by activating the STAT6 signaling pathway and upregulating GATA-3; Directly induces immunoglobulin class switching in B cells to produce IgE; Type 2 T follicular helper (Tfh2) cells are a major source of IL-4, inducing memory B cells to express CD23; Blocking IL-4 or depleting CD4+ T cells inhibits serum IgE production.	6-8, 10, 11
IL-5	Recruits and activates eosinophils, leading to the late-phase reaction and chronic inflammation.	In Nasal Allergen Challenge (NAC) models, elevated IL-5 levels drive eosinophil migration from blood to nasal mucosa; Directly activates eosinophils - anti-IL-5 antibody can inhibit this activation; Persistent activation of the IL-5/eosinophil axis is key to AR transitioning to chronic inflammation; Pharmacological reduction of IL-5 levels significantly reduces eosinophil infiltration and improves clinical symptoms.	12-17
IL-13	Causes mucus hypersecretion and tissue remodeling.	Directly stimulates airway epithelial cells to produce and secrete mucus, particularly MUC5AC; Promotes goblet cell hyperplasia, increasing mucus secretion sources; Acts primarily by activating the STAT6 signaling pathway; Collaborates with eosinophil recruitment and subepithelial fibrosis in IL-13-mediated airway tissue remodeling.	18-22, 24, 26

**Table 2:** Roles of Epithelial Cells and ILC2s in the Innate Immune Axis of Allergic Rhinitis

Cell Type	Key Role	Key Molecules	Specific Mechanisms and Effects	Reference
Epithelial Cells	Immune Sentinels	TSLP, IL-25, IL-33	Respond to allergens (e.g., house dust mites, fungi, pollen) and tissue damage by releasing "alarmins," initiating a type 2 immune response.	28-30
		IL-33	The house dust mite allergen (Der p1) induces its release in a dose- and time-dependent manner.	31
		IL-25	The protease activity and NADPH oxidase activity of pollen play important roles in its release.	33
		TSLP, IL-25, IL-33	Their expression is significantly upregulated in AR and ECRS patients, and their levels positively correlate with clinical severity (e.g., CT scores and eosinophil counts).	30, 31
		TSLP	Nasal epithelial cells in AR patients have intrinsic dysfunction, with baseline mRNA levels significantly higher than in healthy individuals, indicating a "pre-activated" state.	29
		IL-33	Abnormal activation of the upstream Notch-1 signaling pathway can sustain its high expression.	35
		(Various alarmins)	Reduced expression of endogenous protease inhibitors (EPIs) amplifies allergen-induced alarmin release.	34
ILC2s	First Responders	IL-5, IL-13	Directly and rapidly respond to epithelial-derived IL-25, IL-33, and TSLP via surface receptors (e.g., ST2).	36-38
		(Activation)	The activation mechanism is particularly evident in the nasal tissue of AR patients.	39, 40
		(Activation)	In animal models, activation of ILC2s alone, even in the absence of T/B cells, is sufficient to induce nasal inflammation.	41
		(Metabolism)	Upon activation, ILC2s rapidly adjust their metabolism to support efficient effector functions.	42
		IL-5	Primarily responsible for the recruitment and activation of eosinophils.	37, 41
		IL-13	Leads to epithelial hyperplasia and mucus secretion; a key mediator exacerbating nasal epithelial thickening and eosinophil infiltration.	38, 41, 43

**Table 3:** Transcriptional and Signaling Networks Regulating Type 2 Immunity

Pathway/Network	Key Molecules	Function	Regulatory Mechanism	References
IL-4/STAT6/GATA3 Axis	IL-4, IL-4R, CD44v5, STAT6, GATA3, TCF-1, SATB1, Grail, CIS, BLOC1S1, STING, NF-κB	Drives Th2 cell differentiation, initiates transcription of Th2 signature cytokines (IL-4, IL-5, IL-13), inhibits Th1 pathway	IL-4 binds IL-4R; CD44v5 stabilizes IL-4Rα. STAT6 phosphorylation induces GATA3 expression. GATA3 & STAT6 co-regulate Th2 genes. Inhibits suppressive TCF-1 isoforms; Induces SATB1. Negative Feedback: Grail degrades STAT6; CIS inhibits STAT6. BLOC1S1 loss -> mtDNA leakage -> STING-NF-κB activation -> STAT6 phosphorylation	44-52
STAT6-Independent Pathway (Notch)	Notch, Jagged2, KLF2, HIF-1α, Mib1, KLF2/S1PR1 axis	Acts as a "licensing" signal, promotes egress of Th2 cells from draining lymph nodes to effector tissues like the lung, independent of GATA3	"Licensing" signal for Th2 cell egress from lymph nodes. GATA3-independent function. Migration achieved by upregulating KLF2/S1PR1 axis. Regulated by DC signals: KLF2 (via HIF-1α) negatively regulates Jagged2. Mib1 is a necessary molecular switch for ligand activity.	53-55
STAT6-Independent Pathway (IL-2/STAT5)	IL-2, STAT5, C-MAF, Eos, SOCS (CIS, SOCS2)	In human CD4+ T cells, IL-2 via STAT5 induces C-MAF expression, promoting Th2 differentiation; Eos enhances STAT5 activity	IL-2 activates STAT5, directly inducing C-MAF expression. Eos forms a complex with STAT5, enhancing its phosphorylation and activity. Negative Feedback: CIS and SOCS2 inhibit STAT5 and STAT6 phosphorylation.	56-59
GATA3 as Common Core Transcription Factor for Th2 and ILC2s	GATA3, Distal Enhancers (Gata3 +674/762, G3SE, mG900), IL-10, STAT3, Blimp-1, Bcl6, BACH2	Drives the differentiation, maintenance, and function of Th2 cells and ILC2s; regulates expression of type 2 cytokines (e.g., IL-4, IL-5, IL-13)	Regulated by cell-specific enhancers (e.g., G3SE for ILC2s; mG900 for Th2). Regulated by cell-specific upstream TFs: IL-10-STAT3-Blimp-1-Bcl6 axis promotes GATA3 in lung Th2. BACH2: Positive regulator in ILC2s, inhibitory in Th2. GATA3 dosage affects cell plasticity (e.g., skin ILC2s). Expression is independent of auto-positive feedback.	60-67
Negative Regulatory Network of Type 2 Immunity	T-bet, Tregs, BACH2, miR-15/16, DOCK8, PGI₂, IL-37, TSLP, IL-6, SOCS3, sCTLA-4, BAFF	Maintains immune homeostasis, suppresses allergic inflammation; Tregs inhibit activation of Th2 cells and ILC2s	T-bet: Directly inhibits Th2 cell program. Tregs: Function maintained by intrinsic molecules (BACH2, miR-15/16, DOCK8). Treg External "Licensing": PGI₂ activates suppression function. IL-37 increases Treg numbers and IL-10. TSLP maintains Treg lineage stability. IL-6/SOCS3 Pathway: Inhibits IL-2 signaling needed for Th2. sCTLA-4: Suppresses type 1 immunity, permits type 2 immunity. BAFF: Inhibits Treg function, leading to allergy.	68-74, 76, 77

**Table 4:** Key Genetic Variants in Allergic Rhinitis Susceptibility

Gene/Locus	Variant/SNP	Function/Association Description	Reference
ZNF608	Not specified	A key risk gene for house dust mite-induced AR	80
Near CD28	Not specified	Novel locus specific to East Asian populations	81, 82
9q32	Not specified	Novel pleiotropic region	81, 82
10q25.2	Not specified	Novel pleiotropic region	81, 82
CLEC16A	Not specified	Variants confer a decreased risk of AR, playing a protective role	83
Multiple Genes	Not specified	AR shares over 100 risk genes with asthma and eczema	78
Not specified	rs9565267	Associated with the specific trajectory from atopic dermatitis (AD) to AR development	84
HLA region	Not specified	AR is a causal risk factor for food allergy, linked through shared loci in the HLA region	85
GSDMB	Not specified	Gene polymorphisms show a stronger association with asthma and asthma-comorbid AR than with AR alone	79
HLA region	Not specified	One of the strongest susceptibility loci for AR	86
HLA-DQB1, HLA-B	Amino acid variants	Risk signal directly correlated with specific amino acid variants in the peptide-binding pocket, affecting allergen presentation efficiency	86
HLA region	rs7775228	Significantly associated with AR risk in Chinese Han population, with the risk allele linked to increased total serum IgE levels	87
HLA, TYRO3	Not specified	Interaction explored to comprehensively understand the pathogenic mechanism of AR	88
FLG	Loss-of-function (LoF) mutations	The strongest genetic risk factor for AD; increases risk of subsequent AR and asthma by disrupting barrier integrity	89
FLG	Not specified	Mutations primarily lead to early eczema, promoting aeroallergen sensitization, ultimately causing AR and asthma	90
FLG	Not specified	May have a direct effect on rhinitis at age 10 independent of eczema, suggesting a potential role in the nasal mucosal barrier	90
FLG	Not specified	Common LoF mutations in European populations	91
FLG	Not specified	Extremely rare and not significantly associated with disease in Turkish children	92
FLG	Specific mutations	Specific mutation types identified in African American and Saudi populations, associated with AR and other allergic diseases	89, 93
FLG	Not specified	Poor maternal diet during pregnancy confers a risk comparable to the genetic risk of carrying FLG mutations, highlighting the key role of impaired epithelial barrier function	94
TSLP	rs3806933	Significantly associated with AR risk in Korean population; a key genetic risk driving the "atopic march"	98
TSLP	rs2289277	The risk allele [C] significantly increases the likelihood of developing multiple concurrent allergic diseases	99
TLR3	Not specified	Variants directly associated with aeroallergen sensitization; TLR3 signaling can induce TSLP expression	100
IL4R	I50V (rs1805010), Q576R (rs1801275)	Functional missense mutations significantly associated with atopy risk	97
IL4R	rs1801275	Interaction with allergy history analyzed in the context of allergy and childhood leukemia	101
FLG, IL4R	rs3024676	FLG LoF mutations significantly increase the risk of allergic sensitization only in individuals homozygous for the IL4R SNP rs3024676	102
STAT6	rs324011	TT homozygous genotype significantly associated with increased risk of allergic asthma and elevated total serum IgE levels	96
IL33, IL1RL1	Rare variants	Exome sequencing identifies this region as one of the strongest gene clusters associated with asthma and allergy	95
IL33	rs1342326	Variant genotype significantly associated with AR risk in school-aged children, adjusted OR=3.23	103
IL33	rs4742170	The risk 'T' allele disrupts the glucocorticoid receptor (GR) binding site, weakening its suppression of IL33 expression	104
TSLP, IL-33	Not specified	Both play roles in allergic inflammation; TSLP is an important upstream regulator of IL-13	105
FCER1G	rs36233990	Significantly enriched in patients with AR comorbid with asthma, directly linking genetic variation to the core effector phase of AR	106

**Table 5:** Summary of Targeted Biologics for Allergic Rhinitis

Biologic Agent	Target	Mechanism of Action	Key Clinical Findings	Other Characteristics	Reference Citations
Omalizumab	IgE	Binds to free IgE in serum, preventing its binding to the high-affinity receptor (FcεRI) on effector cells, thereby blocking the upstream allergic cascade and downregulating FcεRI expression	Significantly improved nasal and ocular symptoms, quality of life, and work efficiency in severe pollinosis patients inadequately controlled by standard therapy; Real-world studies support efficacy in SAR and PAR, especially for nasal congestion, with long-term safety and efficacy	Effective as pre-seasonal prophylactic treatment; The ratio of total IgE level at week 16 to baseline (≥2.0) may predict clinical response; Patients with more severe baseline symptoms show greater improvement; Reduces work productivity loss; Effective in chronic urticaria; Improved behavioral symptoms in an ASD child	150-161
Dupilumab	IL-4/IL-13	Specifically binds to IL-4Rα, simultaneously blocking signaling of both IL-4 and IL-13	Significantly "normalized" the disease-associated gene expression profile in nasal tissue of AR patients; Reduced serum levels of type 2 inflammatory biomarkers (total IgE, TARC, eosinophils); Shows efficacy in comorbidities like Asthma with AR, CRSwNP with AR, AD with AR	Combined with SCIT improves SCIT tolerability, allows more patients to reach maintenance dose, and reduces epinephrine use; In severe refractory AD, combination with SCIT induced favorable immunological changes	162-173
Tezepelumab	TSLP	Specifically binds and blocks TSLP, broadly inhibiting downstream inflammatory pathways	Significantly and sustainably reduced the annualized asthma exacerbation rate (AAER), improved lung function and quality of life in severe asthma patients, irrespective of baseline Type 2 biomarker levels, with good long-term safety; Effective in subgroups including patients with CRSwNP, PAO, NSAID sensitivity, and Japanese patients	Combination with SCIT enhanced efficacy and showed partially sustained clinical benefit one year after discontinuation, suggesting potential for inducing long-term immune tolerance	174, 175, 177-183
